# A decline of protective apolipoprotein J and complement factor H concomitant with increase in C5a 3 months after cardiac surgery—Evidence of long-term complement perturbations

**DOI:** 10.3389/fcvm.2022.983617

**Published:** 2022-12-20

**Authors:** Krzysztof Laudanski, Da Liu, Damodar Gullipalli, Wen-Chao Song, Tony Okeke, Wilson Y. Szeto

**Affiliations:** ^1^Department of Anesthesiology and Critical Care, The University of Pennsylvania, Philadelphia, PA, United States; ^2^Department of Neurology, The University of Pennsylvania, Philadelphia, PA, United States; ^3^Leonard Davis Institute for Health Economics, The University of Pennsylvania, Philadelphia, PA, United States; ^4^Department of Obstetrics and Gynecology, Shengjing Hospital of China Medical University, Shenyang, China; ^5^Department of Systems Pharmacology and Translational Therapeutics, The University of Pennsylvania, Philadelphia, PA, United States; ^6^Department of Bioengineering, Drexel University, Philadelphia, PA, United States; ^7^Division of Cardiovascular Surgery, Department of Surgery, University of Pennsylvania, Philadelphia, PA, United States

**Keywords:** cardiac surgery, complement, terminal complement complex, complement component 5a, complement factor H, apolipoprotein J, clusterin

## Abstract

**Background:**

Heart surgery results in complement activation with the potential for collateral end-organ damage, especially if the protective elements (complement factor H, Apolipoprotein J) are inadequate. Here, we have investigated if peri-operative stress results in an imbalance between complement activation and its protective mechanisms up to 3 months after heart surgery.

**Methods:**

101 patients scheduled for non-emergent cardiac surgery donated blood before the procedure (t_baseline_), and 24 h (t_24*h*_), 7 days (t_7*d*_) and 3 months (t_3*m*_) after. Complement activation was measured as a serum level of soluble activated component 5 (sC5a) and soluble terminal complement complex (sTCC). Simultaneously, protective complement factor H (CfH), and apolipoprotein J (ApoJ) were measured. Inflammatory responses were quantified using C-reactive protein (CRP) and interleukin-6 (IL-6). Details regarding anesthesia, intensive care unit (ICU) stay, pre-existing conditions, the incidence of postoperative complications, and mortality were collected from medical records.

**Results:**

C5a declined at t_24*h*_ to rebound at t_7*d*_ and t_3*m*_. sTCC was significantly depressed at t_24*h*_ and returned to baseline at later time points. In contrast, CfH and ApoJ were depressed at t_3*m*_. Milieu of complement factors aligned along two longitudinal patterns:cluster#1 (C5a/sTTC continuously increasing and CfH/ApoJ preserved at t_baseline_) and cluster#2 (transient sC5a/sTTC increase and progressive decline of CfH). Most patients belonged to cluster #1 at t_24*h*_ (68%), t_7d_ (74%) and t_3m_ (72%). sTCC correlated with APACHE_1*h*_ (*r*^2^ =−0.25; *p* < 0.031) and APACHE_24*h*_ (*r*^2^ = 0.27; *p* < 0.049). IL-6 correlated with C5a (*r*^2^ =−0.28; *p* < 0.042) and sTTC (*r*^2^ =−0.28; *p* < 0.015). Peri-operative administration of acetaminophen and aspirin altered the complement elements. Prolonged hospital stay correlated with elevated C5a [*t* (78) = 2.03; *p* = 0.048] and sTTC serum levels [*U* (73) = 2.07; *p* = 0.037]. Patients with stroke had a decreased serum level of C5a at t_7d_ and t_3m_.

**Conclusion:**

There is a significant decrease in complement protective factors 3 months after cardiac surgery, while C5a seems to be slightly elevated, suggesting that cardiac surgery affects complement milieu long into recovery.

## Introduction

Complement activation is triggered in three ways: classical, alternative and lectin-driven to activate an innate inflammation to augment pathogens and dead cell removal (1, 2). All pathways merge at C5a and C5b, followed by the formation of the terminal complement complex (TCC) as the common pathway. C5b-9 inserts itself into the cell membrane as one of the primary effector bactericidal mechanisms (3). However, TCC can be self-damaging *via* endothelial activation, cell death, and intravascular hemolysis. Several proteins keep the complement activation in check, with CD55, CD59, and CD46 being the most extensively studied (4, 5). In contrast, humoral factors such as complement factor H and clusterin/Apo J have experienced much less scrutiny despite their critical role in limiting collateral complement-mediated damage (6–19). Their depletion may exacerbate end-organ damage and contribute to an excess of morbidity (10, 13, 15, 16, 20).

During heart surgery, the complement system is activated *via* contact with artificial surfaces, immunoglobulins, or *via* a CRP-mediated pathway (18, 19). As a result, direct complement cytotoxicity is exacerbated by a reperfusion injury and the influx of inflammatory leukocytes (21). Subsequent vasoconstriction, thrombosis, and inflammation may result in hypoperfusion organ injuries. It is not surprising that imbalance in complement has been suggested as the target of therapeutic interventions. However, interference with effector components of complement failed to demonstrate favorable clinical outcomes, except in severely sick patients undergoing heart surgery (22–24). This lack of progress in effectively modulating complement activation may result from neglecting the post-surgical abnormalities in protective elements of complement (22–25). No study addressed the long-term changes in serum mechanisms moderating complement activation after cardiac surgery.

Here, we addressed the knowledge gap in temporal dynamics between cytoprotective (clusterin/ApoJ, complement factor H) vs. cytotoxic (C5a, TTC) elements after non-emergent cardiac surgery. We hypothesized that activation of complement and protective effectors would be synchronized to minimize end-organ damage (16, 17, 26). Specifically, patients with a misalignment between complement effector and protective would experience increased thrombotic events. Finally, we hypothesized that acute disturbances in complement components will resolve 3 months after surgery.

## Patients and methods

### Patients enrollment

Our study protocol was approved by the Institutional Review Board (IRB) of the University of Pennsylvania (#815686). All adult patients scheduled for non-emergent heart surgery were approached for consent. We excluded patients with pre-existing immunological aberrancies on immunosuppressant medications in the last 6 months (prednisone PO or IV more than 5 mg daily, αTNFα, αIL-6, αIL-3, αCD20 antibodies therapy, immunoglobulin, plasmapheresis, methotrexate, chemotherapy). The study did not include patients post-transplant.

### Demographical and clinical data collections

Electronic medical records (EMR) were used to collect demographic and medical data for all enrolled participants. Charlson Comorbidity Index (CCI) measured the burden of chronic disease (27). EMR were extracted for description of surgical procedure and bundle as coronary artery graft bypass (CABG), aortic vavle surgery, mitral valve surgery, aortic arch surgery, and others (Table 1). Most patients have multiple procedure done during one surgery. The perioperative insult was gauged by the duration of anesthesia and surgery, estimated blood loss, and volume of crystalloid resuscitation. The usage of opioids, benzodiazepines, acetaminophen, ketorolac, and steroids during the perioperative 24 h was registered. Acute physiology and chronic health evaluation II (APACHE II) was calculated within 1 h (APACHE_1*h*_), 24 h (APACHE_24*h*_), and 48 h (APACHE_48*h*_) after admission to the Intensive Care Unit (ICU) (28). The severity of the illness was determined by Sequential Organ Failure Assessment (SOFA) (29). Organ failure was defined according to MODS criteria or the Glue Grant framework (30, 31). Depp venous thrombosis (DVT), pulmonary embolism (PE), and stroke diagnoses were extracted from medical records. Survival was determined at 28 days and 3 months. Platelets count was extracted from routine lab from EHR.

**TABLE 1:** Demographical and clinical characteristic of the studied sample.

Demographics (101 patients)	
Age [X ± SD]	62.6 ± 12.44
Over 60 [%]	33.7%
**Gender**	
Male [%]	75.24%
Female [%]	24.75%
Not reported [%]	0%
**Race**	
Hispanic Latino [%]	1.98%
Black [%]	5.94%
White [%]	90.1%
Other/Asian/unknown [%]	3.96
**Pre-existing conditions**	
Weigh	84.6 ± 21.33
BMI	27.6 ± 5.47
Charleston comorbidity index [X ± SD]	3.9 ± 2.03
ACS/MI [%]	13.86%
CHF [%]	15.8%
PVD [%]	9.9%
CVA/TIA [%]	9.9%
Dementia [%]	0%
COPD [%]	5.94%
DM [%]	28.7%
**Anesthesia and surgery data**	
Duration of anesthesia; mean ± SD [min]	372.3 ± 105.89
Duration of surgery; mean ± SD [min]	266.8 ± 101.34
Duration of cardiopulmonary bypass; mean ± SD [min]	129.2 ± 65.04
Coronary artery bypass surgery; no.	51
Mitral valvuloplasty and replacement; no.	26
Aortic valvuloplasty and replacement; no.	40
Aortic aneurysm repair; no.	8
Others; no	4
Estimated blood loss [ml]	201.1 ± 283.89
**Perioperative management**	
***Transfusions during surgery***	
Packed red blood cells, mean (IQ25; IQ75) [mL]	115 [0; 1,200]
Fresh frozen plasma, mean (IQ25; IQ75) [mL]	93.4 [0; 1,750]
Total crystalloid during surgery[mL]	1,252 ± 552.38
***Clinical care during 24 h post-surgery***	
Packed red blood cells, mean; (IQ25; IQ75) [mL]	12.1 [0;600]
Fresh frozen plasma, mean; (IQ25; IQ75) [mL]	9.1 [0; 750]
Corticosteroid administration (% of all cases)	7.9%
Ketorolac administration (% of all cases)	7.9%
Acetaminophen administration (% of all cases)	29.7%
Acetylsalicylic acid administration	32.7%
Opioids administration	689.2 ± 221.89
BZD administration	0.38 + 2.34
**ICU stay**	
APACHE score at 1 h, mean ± SD	16.0 ± 5.55
APACHE score at 24 h, mean ± SD	8.8 ± 4.29
APACHE score at 48 h, mean ± SD	8.4 ± 4.11
**Outcome at 28 days**	
LOS ICU	8.1 ± 40.23
LOS hospital	10.1 ± 21.08
DVT	0.99%
PE	2.97%
CVA	6.93%
Discharged/in the healthcare facility/expired	90.9%/5.94%/2.97%

The characteristics of the studied population are presented in Table 1.

### Sample procurement

Blood was collected in sodium citrate tubes after collection from an arterial or central line. Plasma was isolated by centrifugation for 10 min at 1,200 ×g 4°C, aliquoted, and stored at −80°C. Blood was collected before non-emergent cardiac surgery (t_baseline_) followed by 24 h (t_24*h*_) and 7 days (t_7*d*_) later, with final follow-ups at 3 months (t_3m_).

### Measures of complement effector activation

To detect human C5a levels in plasma samples, αC5a antibody neo-epitope (Biolegend, San Diego, CA) was utilized. Secondary detection was done with biotinylated anti-human C5a mAb (Biolegend, San Diego, CA) and avidin or streptavidin conjugated to horseradish peroxidase (BD, Franklin Lakes, NJ). Recombinant hC5a (Hycult, Wayne, PA) was used as the standard. An analogous process was utilized for the detection of sTCC by utilizing α human TCC mAb neoepitope (SantaCruz, San Diego, CA), biotinylated anti-human TTC mAb (QDC5, in-house). sC5b-9 Complex (Complement Tech, Marlon, NJ) used as standard.

### Assessment of the complement protective factors and inflammation markers

Complement factor H, apolipoprotein J, and C-reactive protein were measured with the multiplex kit (Thermofisher, Waltham MA). IL-6 in serum was determined *via* ELISA (Thermofisher, Waltham MA).

### Statistical analysis

The Shapiro-Wilk W test and distribution plots tested the normality and distribution of variables. Parametric variables are expressed as mean ± SD and compared, using *t*-Student. For non-parametric variables, median (M_*e*_) and interquartile ranges (IR) will be shown with the U-Mann-Whitney statistic, employed to compare such variables. ANOVA was calculated for parametric variables with multiple discrete values, with Shaffe’s test as a *post hoc* test. When applicable, paired contrasts for longitudinal comparisons were used with t_baseline_ as the reference point. Correlational momentum was calculated as *r*^2^ Pearson. A regression analysis was done stepwise methods when appropriate. *k*-means cluster analyses and data normalization were calculated with *scikit-learn* package. A *p*-value less than 0.05 was considered statistically significant for all tests based on the hypothesis. Statistical analyses will be performed with SPSS 26 [IBM, Whalton, NY), and in R (32)].

## Results

Longitudinal analysis of cytotoxic (sTTC, C5a) and protective humoral complement factors (clusterin/ApoJ, factor H) after cardiac surgery.

Age over 60, gender, and race did not significantly affect the baseline levels of studied factors (data not shown).

**FIGURE 1:**
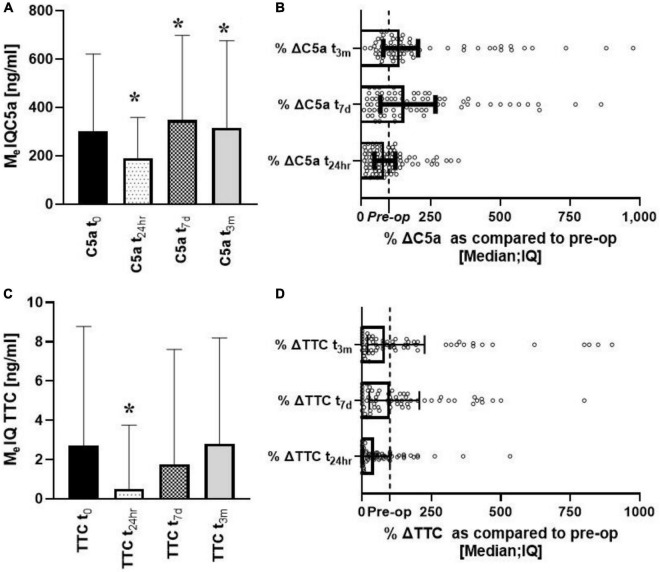
Longitudinal analysis of complement effectors (sC5a, sTCC) after heart surgery. Serum levels of C5a decreased early after surgery to increase up to 3 months of follow-ups **(A,B)**. In contrast, sTTC was depressed only at t_24*h*_ **(C,D)**. *Denotes the statistical significance of 0.05 or less.

sC5a changed significantly over time with concentrations initially decreasing [U (85) =−3.17; *p* = 0.0015), rebounding to significantly higher values at 7 days [U (79) = 2.54; *p* = 0.011] and 3 months (U [69] = 3.34; *p* = 0.00082) (Figure 1A). When data were compared pairwise, the median changes from baseline were 83, 136, and 150% at t_24*h*_, t_7*d*_, and t_3*m*_, respectively (Figure 1B). sTTC levels changed significantly at t_24*h*_ [U (63) =−4.31; *p* = 0.00016] followed by recovery to pre-surgical values (Figure 1C). When the data were compared pairwise, the median changes from baseline were 43, 101, and 83% at t_24*h*_, t_7*d*_, and t_3*m*_, respectively (Figure 1D). However, significant sTTC variability at t_baseline_ and t_3m_ was apparent. The correlation between sTTC and C5a was present only at t_24*h*_ (*r*^2^ = 0.37; *p* < 0.004) (data not shown). A regression analysis revealed that the level of sC5a was the most significant contributor to sTTC levels at t_24*h*_, accounting for 56% (*p* = 0.0067) and 26% (*p* = 0.041) of sTTC variance.

**FIGURE 2:**
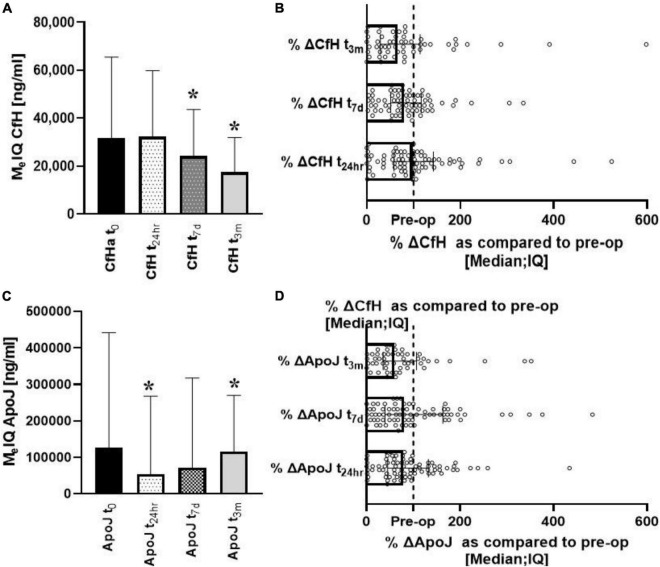
Longitudinal analysis of complement protective factors (CfH, ApoJ) after heart surgery. Progressive depletion of the CfH could be seen in the post-op period **(A,B)**. In contrast, ApoJ started to recover after initial depletion **(C,D)**. *Denotes the statistical significance of 0.05 or less.

Significant changes over time were seen for both CfH and ApoJ. CfH levels were the lowest at t_7d_ [W (70) = 3.06; *p* = 0.002] and t_3*m*_ [W (56) = 3.82; *p* = 0.00013] (Figure 2A). When the data were compared pairwise, the median changes from baseline were 94, 84, and 64% at t_24*h*_, t_7*d*_, and t_3*m*_, respectively, (Figure 2B). ApoJ was lowest at t_24*h*_ [W (86) = 2.56; *p* = 0.01] and at t_3m_ [W (60) = 3.46; *p* = 0.00053] (Figure 2C). The changes from baseline were 81, 82, and 58%, respectively (Figure 2D).

The interplay between complement protective and effector proteins.

**FIGURE 3:**
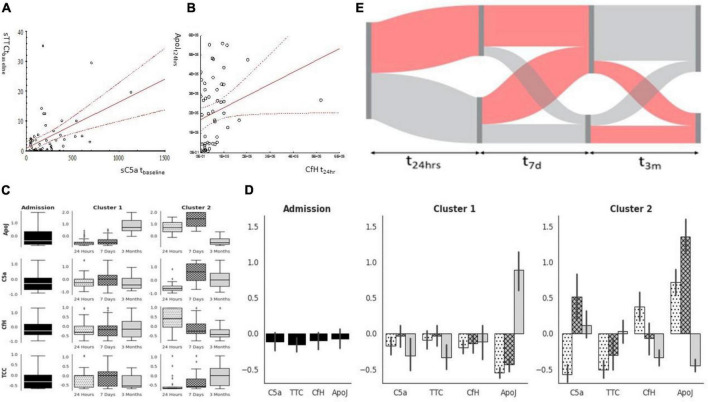
Complement factors milieu after cardiac surgery. C5a and TTC correlated strongly at the baseline **(A)**, while protective factors (ApoJ CfH) correlated at t_24*h*_ **(B)**. While factors were analyzed using clustering, two subgroups emerged: cluster#1 can be characterized by a significant increase in ApoJ at 3 months while cluster#2 is characterized by significant activation of sC5a later after surgery concomitant ApoJ **(C,D)**. Patients’ complement milieu was not stagnant as complement activation characteristics moved between clusters **(E)**.

There was a significant correlation between C5a and sTTC (Figure 3A) and CfH and ApoJ (Figure 3B) at t_24*h*_. The unsupervised analysis identified two clusters with different time dynamics across all four factors studied (Figures 3C,D). Cluster #1 was a cluster with the gradual activation of C5a and sTTC over time while protective factors remained stable (CfH) or increased over time (ApoJ) (Figures 3C,D). Cluster #2 demonstrated an increase in sC5a, sTCC, and ApoJ at 7 days to decline at t_3*m*_ (Figures 3C,D). However, CfH rapidly declined and remained low at t_3m_ in the case of patients in complement cluster#2 (Figures 3C,D). Most patients belonged to complement cluster #1 at t_24*h*_ (68%), t_7*d*_ (74%), and t_3*m*_ (72%). Patients belonged to the original t_24*h*_ cluster, but the transition of individuals to other complement clusters was seen at t_7d_ and t_3m_ (Figure 3E).

### Relationship of complement activation vs. severity of the peri-operative injury

There was no correlation between the time on cardiopulmonary bypass, aortic cross-clamp, or anesthesia and any complement markers (data not shown). sTCC correlated with APACHE_1*h*_ (*r*^2^ = 0.25; *p* < 0.031) and APAPCHE_24*h*_ (*r*^2^ = 0.27; *p* < 0.049). CfH (*r*^2^ = 0.32; *p* < 0.003) correlated with the volume of transfused PRBC during t_24h*r*_. Similar correlations were seen between CfH (*r*^2^ = 0.29; *p* < 0.005), and the volume of transfused fresh frozen plasma was transfused at the same time. Finally, the amount of crystalloid administered correlated weakly with CfH (*r*^2^ = 0.28; *p* < 0.008) during surgery. C5a did not correlate with any measured variables of surgical insult severity.

Serum IL-6 inversely correlated with C5a (*r*^2^ =−0.28; *p* < 0.042) and sTTC (*r*^2^ =−0.28; *p* < 0.015) at t_24*h*_. CRP correlated highly with CfH (*r*^2^ =−0.42; *p* = 0.029) at t_24*h*_.

Perioperative intake of acetaminophen resulted in diminished [21.5 (16.6; 52.3) vs. 41.5 (29.5; 87.9)] serum levels of CfHt_24*h*_ [U (91) = 2.84; *p* = 0.0048] while individuals receiving aspirin had lower serum sTTC at t_24*h*_ [3.5 (0; 6.7) vs. 3.5 (0; 1.7); U (75) = 1.97; *p* = 0.049]. The intake of ketorolac and steroids had no impact on the serum levels of C5a, sTTC, CfH, or ApoJ (data not shown). The number of benzodiazepines given in the first 24 h after surgery correlated significantly with CfH (*r*^2^ =−0.28; *p* < 0.008), and ApoJ (*r*^2^ =−0.23; *p* < 0.028) at t_24*h*_ but not with the perioperative intake of opioids (data not shown).

### Correlations with clinical outcomes

**FIGURE 4:**
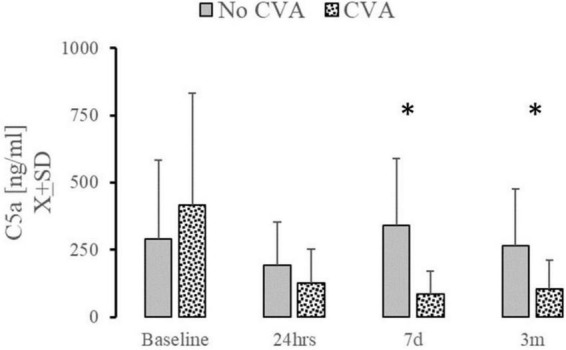
The effect of complement changes on the incidence of cerebrovascular accidents. Serum level of C5a at t_7d_ and t_3m_ was significantly depressed in patients (*n* = 7) who experienced stroke during 28 days after surgery. *Denotes the statistical significance of 0.05 or less.

Low mortality or the incidence of DVT or PE in our studied group precluded the comparison of studied complement factors. Patients who experienced an acute CVA significantly diminished levels of C5a at t_7d_ and t_3m_ (Figure 4). Emergence of AKI at 24 h or at the discharge was not related to changes in C5a, sTTC, CfH, or ApoJ at any time point (data not shown). However, only few patients (*n* = 5) experienced AKI at that time. Patients who were hospitalized at 28 days had significantly elevated serum C5a [186.2 ± 154.3 vs. 343.7 ± 327; *t* (78) = 2.03; *p* = 0.048] and sTTC serum levels [0.3 (0; 3.6) vs. 6 (4.35; 6.9); U (73) = 2.07; *p* = 0.037]. The length of stay (LOS) in the ICU or hospital did not correlate significantly with serum C5a, sTTC, CfH, or ApoJ.

## Discussion

The first unique finding of this study is the observation of severe disruption in the complement milieu extending into post-surgical recovery and outside the period typically considered for peri-operative inflammation. Prior data reported that the effector elements of complement were altered for up to 48 h after surgery (8, 18, 19, 33, 34). Our study focused on 3 months’ performance of complement after heart surgery in adults. We demonstrated increased activity of C5a but no significant changes in serum sTTC in the wake of cardiac surgery and up to 3 months after. C5a plays a vital role in the chemotaxis of granulocytes and in coagulation activation (2, 3). More importantly, elevated C5a increases the risk of graft failure and coronary vasospasm and accelerates atherosclerosis (33, 35, 36). Here we observed decreased level of C5a connected to peri-operative stroke incidence, but the data should be considered a pilot for a more extensive study. The abnormal level of sC5a in patients experiencing a stroke may result from vasculitis consuming complement, as it has been seen in transplanted hearts (36). The etiology of elevated C5a is unclear. Acute inflammation measured by IL-6 seemed resolved. However, elevated mannose levels at the discharge of pediatric patients undergoing heart surgery suggest a potential mechanism of protracted C5a activation (18, 34, 37).

The second important and novel finding is that the long-term increase of sC5a is not counterbalanced by factors protecting from the overactivation of the complement system (6, 11–13, 15, 17, 20). As demonstrated both in time series and cluster analysis, both ApoJ and CfH were severely depressed in several patients if their C5a and sTCC were elevated. This is the first observation of this nature to date. Hemodilution is unlikely to be responsible for decline of ApoJ and CfH considering that 3 months after surgery patients fluid status should be balanced. Excessive consumption is potential reason but a generalized phenomenon like disseminated intravascular coagulation should resolve at 3 months. Also, the platelets count normalized at the time of discharge (data not shown). Interestingly, the milieu of patients’ complement factors created two relatively uniform clusters initially right after surgery. However, over time, patients tended to group in cluster#1. This cluster sustained activation of C5a and preserved ApoJ and CfH, and resembles a natural resolution of complement activation where elevated levels of C5a and sTTC are counterbalanced by protective elements. The alternative cluster was different mainly due to the profound long-term depletion of CfH and ApoJ. CfH is critical as the alternative activation pathway of the complement system (2, 6, 11). Alternative and mannose-driven complement activation is predominantly affected during cardiopulmonary bypass surgery (6, 18, 35). The liver is one of the predominant producers of CfH and ApoJ, but incidence of liver failure was low in our studied population (38). CfH is activated by pentraxin, but this protein family was not in our study (17). Finally, acute inflammation was measured *via* IL-6 and normalized at 7 days so the ongoing inflammatory process cannot account for the decrease in ApoJ and CfH (8, 18).

Applying the multidimensional approach to data is an alternative to the prior studies. It aligns well with the current understanding of critical care illnesses, such as dysregulation failure. It offers a holistic assessment of complement where several biological components are considered as several regulatory components like CfH and ApoJ may be critical in restoring post-cardiac effector components imbalance (22, 23). It also offers a new approach for treating the post-cardiac surgery abnormalities as the prior clinical studies modulating complement failed to demonstrate widespread clinical benefit (25). Anti-complement modulators have been advocated for long time as potential drugs for patient undergoing heart surgery but clinical trial failed. The failure is often attributed to insufficient understanding of complement activation during heart surgery (39, 40). Our study suggested that complement performance is multidimensional and involved multiple pro- and anti-complement factors (2, 22). It is possible that modulating of C5a/sTTC vs. supplementing ApoJ or CfH needs to be precisely targeted in specific patients subpopulation as demonstrated in our cluster analysis. The clinical meaning of our findings has yet to be determined as limited study samples, and incidence of stroke precluded statistical analysis.

Limitations of our study need to be acknowledged. First, extending the study to a different center and using commercially available kits is necessary to generalize the findings. The high standardization of care in our system may have increased the chance of this bias. Second, we only investigated the effector arm of the complement activation system while interference with the mannose/lectin activated pathway may be more dominant in cardiac surgery patients (34, 41). Other elements of the complement regulatory components were not measured while representing distinctive activation, inhibition, and regulation (4, 12, 17, 26, 37). Third, our study was not powered to look for the clinical impact of the complement milieu. No prior study has analyzed several complement factors simultaneously. Most of the correlations between complement factors and clinical measurements reflecting cardiac surgery severity were weak and, at best, suggested potential relationships. Most of these correlations were related to blood loss or perioperative transfusions. Consequently, changes in the complement system may be related to exogenous injections of CfH and ApoJ as they are abundant in serum (6, 7). Finally, CfH polymorphisms is rare and unlikely as the factor affecting results (14). Also, pre-existing condition leading to surgery were very ambiguous. Some patients had diagnosis of coronary artery disease, while others had pre-existing endocarditis leading to surgery. Several others pre-existing conditions could be extracted from chart. Several of these conditions may affect the baseline complement status at baseline with even less predictable effect for the peri-surgical fluctuations of factors (2, 22, 36).

Our pilot study offers several methodological advantages over prior studies. First, it provides a relatively large sample and, more importantly, a longitudinal analysis extending 3 months after surgery (18). The same heparinized circuit was used throughout the study’s duration (19). We also account for the acetaminophen, ketorolac, aspirin, and steroids used during the surgery (19). High standardization of the care reduced post-surgery care variability. We accounted for inflammation levels by measuring IL-6. Several components of perioperative management were factored in more detail as compared to prior studies (18, 22, 25).

In summary, we demonstrated for the first time imbalance between C5a and CfH and ApoJ three months after non-emergent cardiac surgery. This imbalance was related to the longer LOS and emergence of cerebrovascular events.

## Data availability statement

The raw data supporting the conclusions of this article will be made available by the authors, without undue reservation.

## Ethics statement

The studies involving human participants were reviewed and approved by the University of Pennsylvania IRB. The patients/participants provided their written informed consent to participate in this study.

## Author contributions

KL: conceptualization, methodology, investigation, and writing—original draft preparation. KL and TO: formal analysis, data curation, and visualization. KL, TO, W-CS, DG, and WS: writing—review and editing. KL and WS: project executions and funding acquisition. All authors have read and agreed to the published version of the manuscript.
